# Gastrectomy-induced alterations in gut microbiota linked to changes in oral and gastric microbiota

**DOI:** 10.3389/fmicb.2025.1599503

**Published:** 2025-06-18

**Authors:** Eri Komori, Nahoko Kato-Kogoe, Yoshiro Imai, Shoichi Sakaguchi, Kohei Taniguchi, Michi Omori, Mayu Ohmichi, Wataru Hamada, Shota Nakamura, Takashi Nakano, Sang-Woong Lee, Takaaki Ueno

**Affiliations:** ^1^Department of Dentistry and Oral Surgery, Faculty of Medicine, Osaka Medical and Pharmaceutical University, Takatsuki, Osaka, Japan; ^2^Department of General and Gastroenterological Surgery, Faculty of Medicine, Osaka Medical and Pharmaceutical University, Takatsuki, Osaka, Japan; ^3^Department of Microbiology and Infection Control, Faculty of Medicine, Osaka Medical and Pharmaceutical University, Takatsuki, Osaka, Japan; ^4^Division of Translational Research, Center for Medical Research and Development, Osaka Medical and Pharmaceutical University, Takatsuki, Osaka, Japan; ^5^Department of Infection Metagenomics, Genome Information Research Center, Research Institute for Microbial Diseases, Osaka University, Suita, Osaka, Japan

**Keywords:** gastrectomy, gut microbiota, oral microbiota, gastric microbiota, 16S rRNA, gastric cancer

## Abstract

**Introduction:**

Gastrectomy serves as a primary treatment for gastric cancer, a leading global malignancy, and affects significant physiological and anatomical changes in the digestive tract. Recent studies highlight the critical role of gastrointestinal microbiota in postoperative health following digestive tract surgeries, including gastrectomy. These alterations possibly impact the gut microbiota and affect patient health by influencing the bacterial environment in the gastrointestinal tract. However, the relationships between the gastrointestinal tract and the oral, gastric, and gut microbiota after gastrectomy are not clear. In this study, we aimed to characterize alterations in the gut microbiota due to gastrectomy and evaluate whether these alterations are associated with the oral and gastric microbiota.

**Methods:**

Saliva, gastric fluid, and stool samples were collected from patients diagnosed with primary gastric cancer who underwent gastrectomy at two time points, before and 6 months after gastrectomy. Next, 16S rRNA metagenomic analysis was performed. Diversity and linear discriminant analysis effect size (LEfSe) analyses of each microbiota were conducted before and after gastrectomy to compare alterations in the gut, oral, and gastric microbiota.

**Results:**

The diversity of gut microbiota increased after gastrectomy compared to that before gastrectomy (Shannon index, *p* = 0.044), with LEfSe analysis showing increased abundance of *Rothia* and *Lactobacillus* in the gut microbiota. Additionally, the proportion of participants with *Rothia* in their gut microbiota increased, and this genus was present in the oral and gastric microbiota of almost all participants. Furthermore, a significant rise in *Lactobacillus* was observed in the gut, oral, and gastric microbiota of paired participants.

**Discussion:**

We characterized gut microbiota alterations caused by gastrectomy and demonstrated their relationship with changes in oral and gastric microbiota, thereby elucidating interactions between the gastrointestinal tract microbiota in response to changes in the gastric environment.

## 1 Introduction

Gastrectomy is a key treatment for gastric cancer, one of the most common malignancies worldwide, and leads to significant physiological and anatomical changes in the digestive tract. Gastrointestinal microbiota is closely related to the postoperative health of patients who have undergone digestive tract surgery (Guyton and Alverdy, 2017; Tsigalou et al., 2023). Increasing knowledge of the microbiota has revealed that the relative abundance of bacteria varies depending on the living environment and disease, indicating the importance of maintaining homeostasis (Gao et al., 2018; Shanahan et al., 2021).

The gastrointestinal tract is inhabited by numerous bacteria (Iebba et al., 2016; Sender et al., 2016). Initially, the stomach was considered sterile. However, many bacteria, including *Helicobacter pylori*, that form the gastric microbiota have now been identified (Marshall and Warren, 1984; Bik et al., 2006; Ianiro et al., 2015). Gastric microbiota plays an important role in gastric homeostasis and is altered in patients with gastric cancer (Yang et al., 2021; Mendes-Rocha et al., 2023), including those who have undergone surgical treatment (Tseng et al., 2016; Lin et al., 2018). We previously evaluated the gastric microbiota of patients undergoing distal gastrectomy for gastric cancer and observed a decrease in diversity and a change in bacterial composition 6 months after surgery compared to before surgery (Imai et al., 2022).

Changes in the gastric environment also affect the gut microbiota. For example, bariatric surgery and long-term administration of proton pump inhibitors change the gut microbiota (Gutiérrez-Repiso et al., 2021; Kiecka and Szczepanik, 2023; Xiao et al., 2024). Gastrectomy also affects the gut microbiota (Erawijantari et al., 2020; Maksimaityte et al., 2021); however, most of these reports are from cross-sectional studies comparing healthy subjects. Longitudinal studies comparing the microbiota before and after surgery are scarce, highlighting a need for further research in this area.

The oral cavity is inhabited by more than 700 species of bacteria that form the oral microbiota and are associated with health and disease (Yamashita and Takeshita, 2017; Tian et al., 2024). Recently, oral bacteria have been shown to influence the gut environment (Lu et al., 2023; Tan et al., 2023; Kunath et al., 2024). The proposed mechanisms for oral bacteria to reach the gut are the enteral, hematogenous, and immune cell migration routes. Accumulating evidence suggests that oral bacteria affect the bacterial environment in the guts of healthy individuals; however, no consensus has been reached (Schmidt et al., 2019; Rashidi et al., 2021). Oral bacteria is likely to survive in the gut and act in some way; however, the details remain unclear (Tan et al., 2023; Kunath et al., 2024). In patients with specific diseases, such as inflammatory bowel disease and colorectal cancer, bacteria of oral origin increase in the gut and are associated with pathological conditions (Atarashi et al., 2017; Kitamoto et al., 2020; Yamazaki and Kamada, 2024). Because oral bacteria have been shown to translocate into the gut under certain conditions and are involved in disease, oral and gastric microbiota may be associated with alterations in the gut microbiota owing to gastrectomy. However, this requires verification. For the health of post-gastrectomy patients, a comprehensive evaluation of the gastrointestinal microbiota as a whole is necessary and not just an assessment of the microbiota at individual sites.

In a previous study, we characterized changes in the oral microbiota of patients with gastric cancer who underwent gastrectomy and reported that these changes were associated with changes in the gastric microbiota (Komori et al., 2023). We hypothesized that gastrectomy would facilitate the inhabitation of oral and gastric bacteria in the intestine, which would be associated with changes in the gut microbiota. Therefore, in the present study, we aimed to characterize changes in the gut microbiota of these patients and evaluate their association with alterations in oral and gastric microbiota.

## 2 Materials and methods

### 2.1 Participants

The participants were patients with primary gastric cancer who underwent distal gastrectomy followed by either B1 or RY reconstruction. The procedures were performed in accordance with the Japanese Guidelines for the Treatment of Gastric Cancer at the Department of General and Gastroenterological Surgery, Osaka Medical and Pharmaceutical University Hospital, Takatsuki, Japan, between January 2019 and February 2021. The exclusion criteria were as follows: patients with macroscopic residual disease at the time of surgery (R2 resection); those who received neoadjuvant chemotherapy; those who underwent simultaneous resection of other organs for malignant disease; those with pyloric stenosis; those taking immunosuppressive drugs, corticosteroids, antacids, and antibiotics for at least 3 months before specimen collection; those with infection or autoimmune disease; those undergoing treatment for infectious or autoimmune diseases, renal or hepatic failure, or malignant tumors. The diet or lifestyle of the participants was managed within the scope of usual medical care. In addition, when data were missing, they were excluded from the analysis. A flowchart of participant selection is presented in Supplementary Figure 1.

The study was conducted in accordance with the Declaration of Helsinki and its latest amendments and was approved by the Ethics Committee of Osaka Medical College, Takatsuki City, Japan (approval no. 2145). Written informed consent was obtained from all patients included in the study.

### 2.2 Sample collection

The following samples were collected from the same participants at two time points, before and 6 months after gastrectomy: saliva for oral microbiota analysis, gastric fluid for gastric microbiota analysis, and stool for gut microbiota analysis. Some of the saliva and gastric fluid samples used in this study were part of our previously reported samples (Komori et al., 2023). The saliva and gastric fluid samples were collected as previously described (Omori et al., 2021; Imai et al., 2022). Briefly, the saliva samples were collected in the morning after an overnight fast using the saliva collection system SalivaBio^®^ Oral Swab and Swab Storage Tube (Salimetrics, State College, PA, USA). Thereafter, gastric fluid samples were collected during upper gastrointestinal endoscopy. The samples were stored at −80°C after collection until DNA extraction. Stool samples were collected using Mykinso^®^ fecal collection kits containing guanidine thiocyanate solution (Cykinso, Tokyo, Japan), transported at room temperature, and stored at 4°C.

### 2.3 DNA extraction, 16s rRNA sequencing, and taxonomic classification

DNA extraction, 16S rRNA sequencing, and taxonomic classification were performed using our previously described protocol (Omori et al., 2021). Briefly, DNA was extracted from the saliva, gastric fluid, and stool samples using an automated system (GENE PREP STAR PI-480; Kurabo Industries Ltd., Osaka, Japan), according to the manufacturer's instructions. The V1–V2 region of the 16S rRNA gene was amplified, and sequencing libraries were prepared to generate 250 bp paired-end reads for 500 cycles using the MiSeq Reagent Kit v2, following the 16S metagenomic sequencing library preparation protocol provided by Illumina (San Diego, CA, USA). The obtained sequences were demultiplexed, yielding an average of 40193 sequence reads with 250-bp paired-ends, then denoised and quality filtered using QIIME2 (Quantitative Insights into Microbial Ecology 2) version 2023.05. After quality filtering, 904,082 sequences were obtained, with a mean of 29,149 sequences per sample (min: 16,427; max: 56,531). To set a rarefaction minimum depth value of 10,000 as the cutoff value, all samples were preserved for downstream analysis. Taxonomies were assigned to the final sequences using the Silva 138 reference database.

### 2.4 Statistical analysis

To assess the diversity of the gut microbiota within individuals, the alpha diversity indexes, namely the observed operational taxonomic unit (OTU) index and Shannon's index, were calculated. The Kruskal–Wallis test was used for comparisons between the groups, with statistical significance set at *p* < 0.05. To evaluate differences in the gut microbiota between individuals, beta diversity indexes and unweighted and weighted UniFrac distances were calculated. Principal coordinate analysis was performed using UniFrac distance to visualize differences in the gut microbiota between patients. Compositional differences between before- and after-gastrectomy groups were assessed using permutational multivariate analysis of variance (PERMANOVA). These analyses were performed using Qiime2 version 2023.05. Linear Discriminant Analysis (LDA) scores represented the differential bacterial taxa between the two groups. Linear discriminant analysis effect size (LEfSe) was calculated using the LDA scores. All analyses were performed using the α parameter of LEfSe for pairwise tests set at 0.05, while the LDA score was cut off at 2.0. The relative abundances of specific bacterial genera in the same patients were compared before and after gastrectomy using the Wilcoxon signed-rank sum test, with statistical significance set at *p* < 0.05. All analyses were performed using JMP Pro 17.2.0 (SAS Institute Inc., Cary, NC, USA).

## 3 Results

### 3.1 Participant characteristics

General conditions, gastric cancer status, and oral conditions of the participants for microbiota analysis (*n* = 15) are shown in Table 1. There were no significant differences in BMI or oral status, including the number of teeth or severity of periodontal disease, before and after gastrectomy.

**Table 1:** Characteristics of the study population (*n* = 15).

**General conditions**	
Age (years) (median [min–max])	70 [55–78]
Sex (M/F)	9/6
Body mass index (kg/m^2^): before/after gastrectomy (mean ± SD)	22.7 ± 2.7/21.0 ± 2.4
Never smoker/ex-smoker/current smoker	9/3/3
Dyslipidemia^a^	2 (13.3%)
Hypertension^b^	7 (46.7%)
Diabetes^c^	2 (13.3%)
**Gastric cancer status**	
Clinical stage: (I/II/III/IV)	11/2/2/0
Surgical approach (laparoscopic/open/robotic)	14/1/0
Reconstructive methods after gastrectomy (B1/RY)	10/5
**Oral conditions**	
Number of teeth (median [min–max])	18 [0–28]
Severe periodontitis^d^	2 (13.3%)

B1, Billroth 1; RY, Roux-en-Y.

^a^Dyslipidemia is defined as LDL-C > 140 mg/dl, HDL-C < 40 mg/dl, and triglycerides > 150 mg/dl or the use of anti-dyslipidemic drugs.

^b^Hypertension is defined as blood pressure > 140/90 mmHg or the use of anti-hypertensive drug.

^c^Diabetes is defined as HbA1c > 6.5% (NGSP) or the use of oral anti-diabetic drugs or insulin therapy.

^d^Criteria for staging according to the CDC–AAP case definitions for surveillance of periodontitis (Eke et al., 2012).

### 3.2 Gut microbiota composition before and after gastrectomy

**Figure 1:**
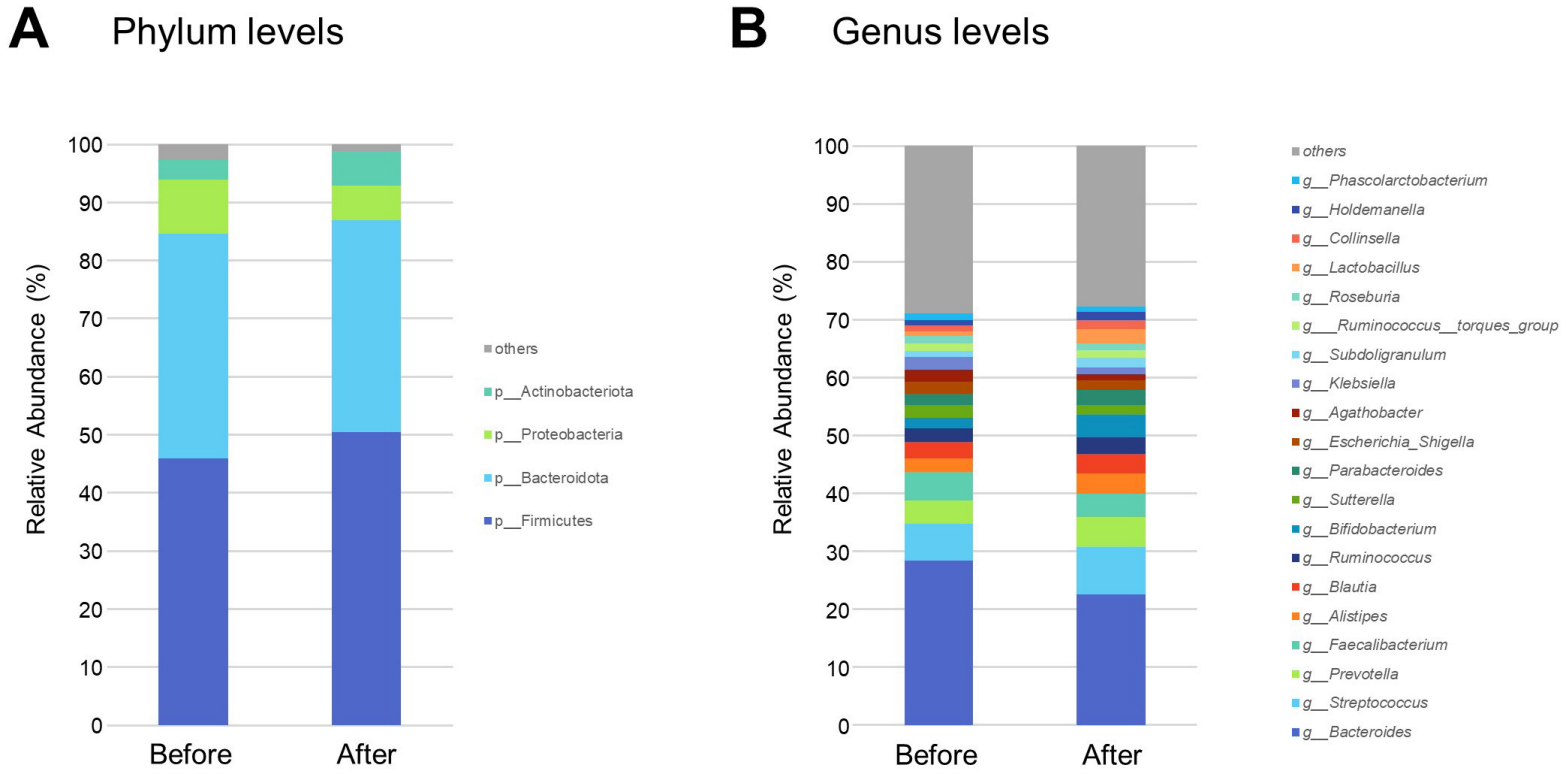
Taxonomic composition of gut microbiota before and after gastrectomy. Vertical bar plots show the relative abundance of bacteria at the phylum **(A)** and genus levels **(B)** before and after gastrectomy. The relative abundances of the four most common phyla and 20 most common genera are shown.

Gut bacteria with a mean relative abundance of at least 0.1% in both before- and after-gastrectomy groups were sorted into 6 phyla, 10 classes, 19 orders, 32 families, and 72 genera. The most abundant bacteria, consisting of more than 5% of the total sequences in either group, belonged to the phyla Bacillota, Bacteroidota, Proteobacteria, and Actinobacteriota, which comprise 97.28 and 98.87% of the gut microbiota in the before and after gastrectomy groups, respectively (Figure 1A). At the genus level, a total of 121 genera were present in at least 30% of the participants in both groups, 97 of which were common between them. In the before-gastrectomy group, 107 genera were found in more than 30% of patients, 10 of which were found in only < 30% of patients in the after-gastrectomy group. In the after-gastrectomy group, 111 genera were found in more than 30% of patients, 14 of which were found in only < 30% of patients in the before-gastrectomy group. The 20 most abundant genera in the before- and after-gastrectomy groups represented 71.11% and 72.31% of the total genus abundance, respectively (Figure 1B).

### 3.3 Differences in gut microbiota before and after gastrectomy

**Figure 2:**
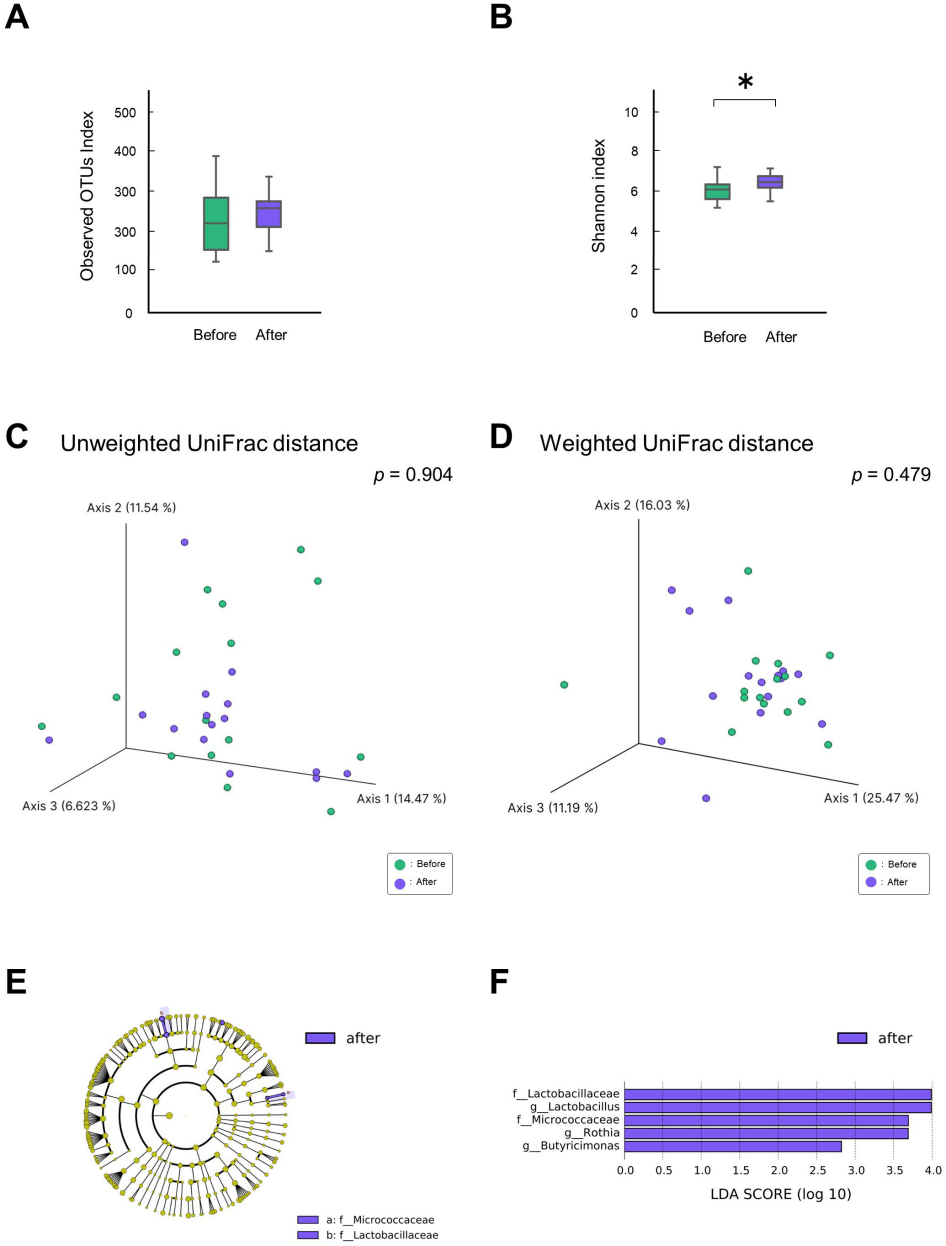
Differences in gut microbiota before and after gastrectomy. Alpha-diversity of the gut microbiota, observed operational taxonomic unit (OTU) index **(A)**, and Shannon index **(B)** in the before- (*n* = 15, green) and after-gastrectomy (*n* = 15, purple) groups. *p < 0.05, compared among groups using the Kruskal–Wallis test. Beta-diversity of the gut microbiota; unweighted **(C)**, and weighted UniFrac distances **(D)**. Principal coordinate analysis (PCoA) plots for samples from 15 participants in the before (green) and after-gastrectomy (purple) groups. Differentially abundant bacterial genera between the before- and after-gastrectomy groups were identified using linear discriminant analysis effect size (LEfSe). Cladograms of differentially abundant bacterial taxa, with each layer representing a different taxon, are shown **(E)**. The enriched taxa in the after-gastrectomy group (purple) are presented in the cladogram but were not found in the before-gastrectomy group. Histogram of linear discriminant analysis (LDA) scores for differentially abundant bacterial taxa between the before- and after-gastrectomy groups **(F)**. LDA scores ≥ 2.0 are shown. Purple represents significantly more abundant taxa in the after-gastrectomy group than that in the before-gastrectomy group.

Analysis of alpha diversity in the gut microbiota showed no significant difference in species richness (observed OTU index, *p* = 0.330; Figure 2A), whereas the evenness in the after-gastrectomy group increased significantly compared to that in the before-gastrectomy group (Shannon index, *p* = 0.044; Figure 2B). Analysis of beta diversity showed no significant differences between the before- and after-gastrectomy groups based on the unweighted (PERMANOVA, 999 permutations, *p* = 0.904, Figure 2C) and weighted (PERMANOVA, 999 permutations, *p* = 0.479, Figure 2D) UniFrac distances.

Specific bacteria that differed in relative abundance from the phylum to genus level between the groups before and after gastrectomy were identified using LEfSe analysis. At the family level, *Micrococcaceae* and *Lactobacillaceae* were relatively abundant, while at the genus level, *Rothia* and *Lactobacillus* were more abundant in the after-gastrectomy group than in the before-gastrectomy group (Figures 2E, F). These results reveal that specific bacteria increased in the gut microbiota of patients after gastrectomy.

### 3.4 Number of bacterial genera shared by the gut, oral, and gastric microbiota

**Figure 3:**
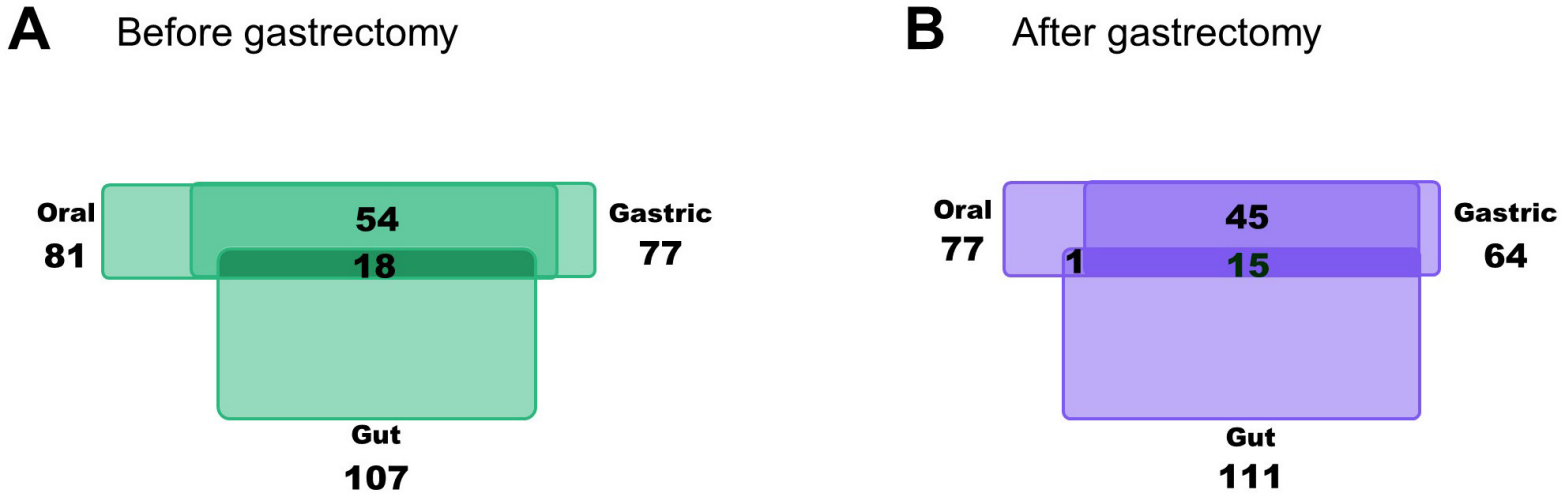
Number of bacterial genera shared by the gut, oral, and gastric microbiota. Based on the analysis of the gut, oral, and gastric microbiota before **(A)** and after **(B)** gastrectomy, information on the number of bacterial genera detected in >30% participants is shown in the Venn diagram.

As a preliminary step to evaluate whether bacteria were altered in the gut microbiota owing to gastrectomy were present in the oral and gastric microbiota, the number of bacterial genera shared by the gut, oral, and gastric microbiota before and after gastrectomy in >30% of the participants were assessed. As shown in Figure 3, the number of bacterial genera present in the gut microbiota increased slightly from 107 to 111 after gastrectomy. In the oral microbiota, the number decreased from 81 to 77, and in the gastric microbiota, it decreased from 77 to 64. Overall, the number of shared bacterial genera among the three microbiota decreased from 18 to 15 after gastrectomy.

**Figure 4:**
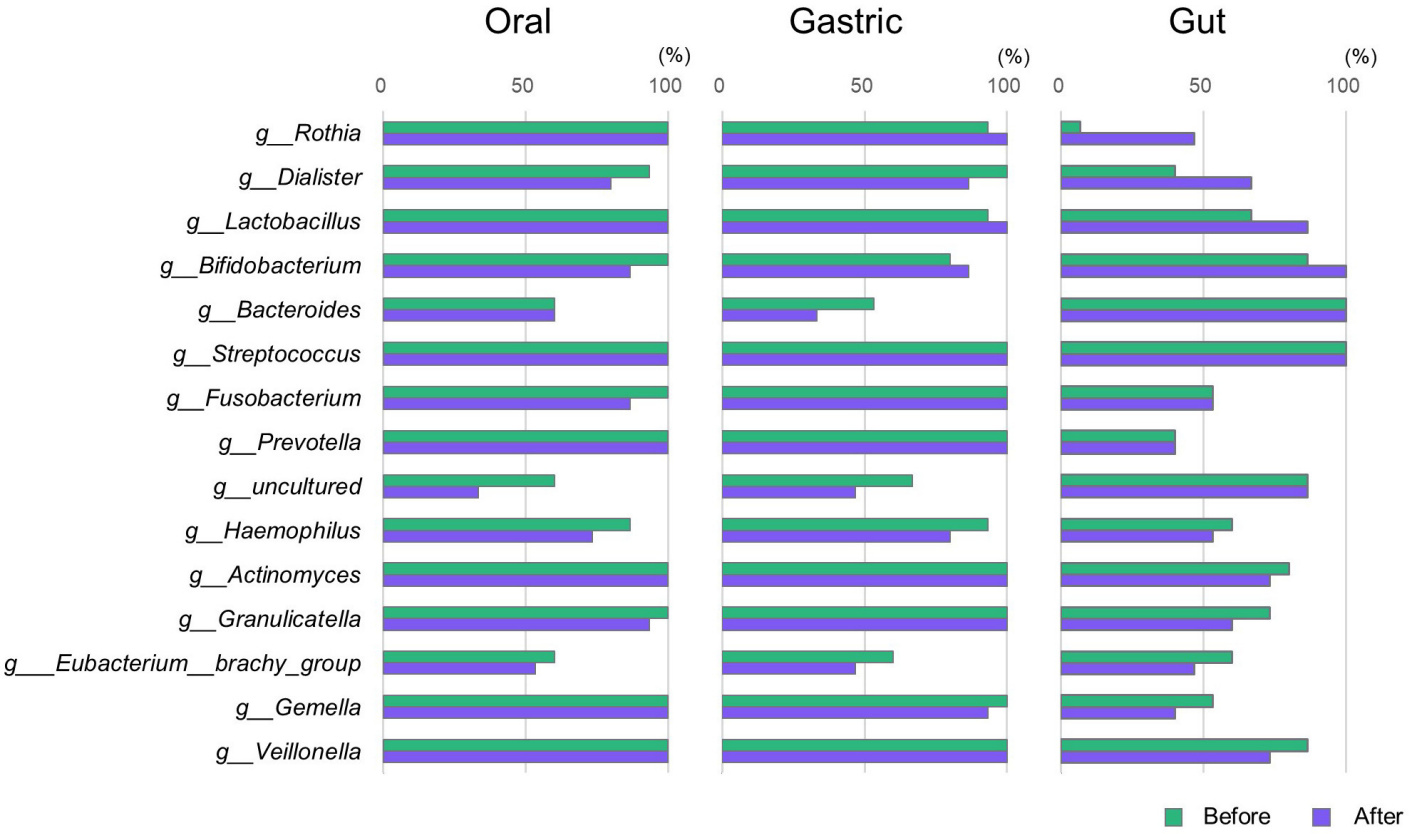
Proportion of participants possessing specific bacterial genera. For the 15 bacterial genera shared by the oral, gastric, and gut microbiota after gastrectomy shown in Figure 3B, the bar graph displays the proportion of participants with each genus in the oral, gastric, and gut microbiota before and after gastrectomy (*n* = 15).

Subsequently, the 15 bacterial genera shared after gastrectomy in the gut, oral, and gastric samples were further evaluated. These 15 shared bacterial genera (Figure 4) included *Lactobacillus* and *Rothia*, which were shown to increase in abundance after gastrectomy compared to those before gastrectomy in an LEfSe analysis of the gut microbiota (Figure 2F).

### 3.5 Alterations in the proportion of participants possessing specific bacterial genera before and after gastrectomy

For the 15 bacterial genera mentioned in Section 3.4, changes in the proportion of participants possessing each genus before and after gastrectomy were examined for the oral, gastric, and gut microbiota (Figure 4). The proportion of participants possessing *Rothia* in the gut microbiota increased after gastrectomy (6.7%−46.7%), and this genus was present in the oral and gastric microbiota of almost all participants before and after gastrectomy. The proportion of participants possessing *Dialister* in the gut microbiota also increased after gastrectomy (from 40.0% to 66.7%), and this bacterial genus was present in the oral and gastric microbiota of ~90% of participants. Furthermore, the proportion of participants with *Lactobacillus* was slightly increased in both the gut and gastric microbiota, which was present in the oral microbiota of all participants.

### 3.6 Alterations in relative abundance of specific bacterial genera before and after gastrectomy

**Figure 5:**
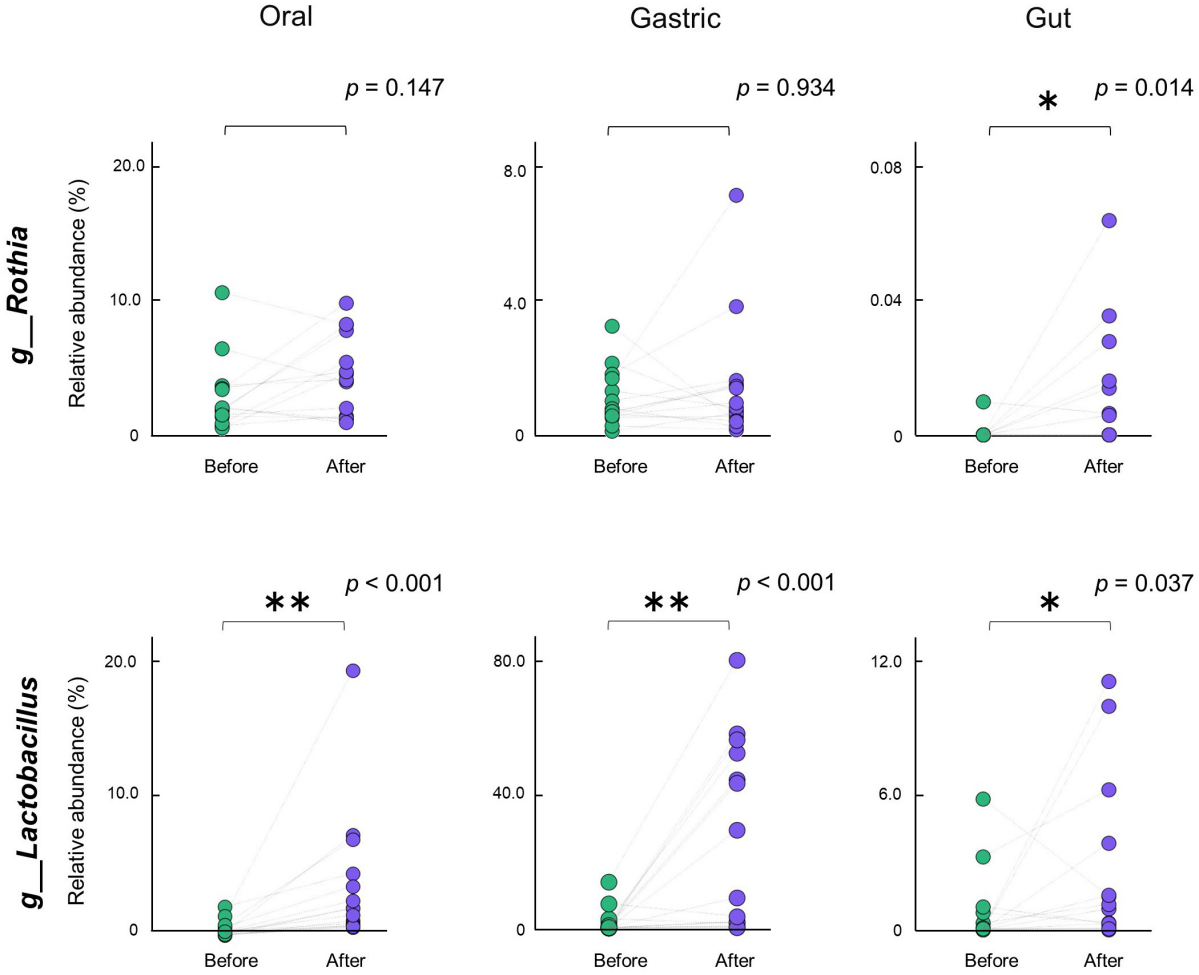
Alterations in relative abundance of specific bacterial genera. Alterations in the abundance of *Rothia, Lactobacillus* in the respective oral, gastric, and gut microbiota before and after gastrectomy of the same participant pair (*n* = 15) are shown. **p < 0.01; *p < 0.05. Comparisons between groups were performed using the Wilcoxon signed-rank sum test.

Next, we examined changes in relative abundance of the 15 bacterial genera shared by the oral, gastric, and gut microbiota after gastrectomy. Specifically, we analyzed whether their abundance shifted before and after gastrectomy in paired participants (Figure 5). In the gut microbiota, similar to the results of the LEfSe analysis, the abundance of *Rothia* and *Lactobacillus* significantly increased after gastrectomy (*p* = 0.014 and *p* = 0.037, respectively). However, the increased abundance of *Rothia* in the oral and gastric microbiota was not significant. The abundance of *Lactobacillus* was significantly increased in the oral (*p* < 0.001) and gastric microbiota (*p* < 0.001).

## 4 Discussion

In this study, we characterized gut microbiota changes following gastrectomy for gastric cancer using 16S rRNA metagenomic analysis. Post-gastrectomy, gut microbiota diversity increased, and certain bacterial genera with significantly elevated abundance were identified. These genera, also present in the oral and gastric microbiota, showed parallel changes across all three sites. Therefore, our data suggest an association between alterations in the gut microbiota owing to gastrectomy and the oral and gastric microbiota.

Following gastrectomy, the diversity of the gut microbiota increased, with LEfSe analysis showing increased abundance of *Rothia* and *Lactobacillus*. Several cross-sectional clinical studies have investigated the composition of gut microbiota in patients after gastrectomy for gastric cancer (Maksimaityte et al., 2021). According to a previous report, patients after gastrectomy have a higher diversity of gut microbiota and a higher abundance of *Lactobacillus* than healthy subjects (Erawijantari et al., 2020). Interestingly, this report is consistent with the results of our longitudinal study. Because the changes in microbiota over time after gastrectomy are not known, in this study, we evaluated changes in microbiota 6 months after gastrectomy, when clinically, the changes in systemic status owing to surgery were normalized. Further follow-up is needed over a longer period. In recent years, changes in the gut microbiota following surgery of the gastrointestinal tract have been implicated in postoperative complications (Guyton and Alverdy, 2017; Tsigalou et al., 2023). Therefore, further elucidation of specific bacteria in the gut of patients undergoing gastrectomy in the future may aid in predicting and preventing the risk of developing complications.

In this study, the proportion of participants possessing bacteria such as *Rothia* spp. in their gut microbiota increased after gastrectomy. The bacteria were present in the oral and gastric microbiota of many participants. This can be attributed to the removal of some of the physical barriers that normally prevent the passage of oral bacteria into the gut due to gastrectomy, and an increase in stomach PH facilitated by the movement of oral bacteria into the stomach and gut. Increased oral bacteria in the gut microbiota may be clinically important, as there is a growing recognition that it may be associated with adverse outcomes in a variety of systemic diseases, including cardiovascular diseases (Li et al., 2024), metabolic disorders (Acharya et al., 2017), autoimmune diseases (du Teil Espina et al., 2019), and inflammatory bowel diseases (Atarashi et al., 2017). *Rothia* spp. are commensal bacteria found in the oral cavity and not normally found in the gut; however, they have been detected in the guts of long-term users of proton pump inhibitors (Imhann et al., 2016). To date, the clinical role of *Rothia* spp. in the gut is not well understood, and further analysis to determine its function would be worthwhile.

In this study, the abundance of *Lactobacillus* spp. significantly increased in the gut, oral, and gastric microbiota of the same participants paired before and after gastrectomy. These results emphasize the close relationships among the gastrointestinal microbiota. The major cause of *Lactobacillus* spp. enrichment is likely a decrease in gastric acid production caused by the surgery. *Lactobacillus* spp. may grow more than other bacteria in the stomach because it is acid tolerant and can proliferate under mildly acidic conditions. Many strains of *Lactobacillus* spp. have garnered attention owing to their probiotic functions. Notably, these strains are predominant in the gastric microbiota of gastric cancer patients and the gut microbiota of colorectal cancer patients (Li et al., 2021; Marashi et al., 2024). Because the genus *Lactobacillus* spp. contains more than 100 species with varied functions, further analysis at the species and strain level is necessary to elucidate its clinical role.

The results of this study showed an association between oral microbiota and alterations in the gut microbiota caused by gastrectomy. Periodontitis, one of the most common oral bacterial infections, is a major risk factor for infectious complications after gastrointestinal surgery, including gastrectomy for gastric cancer (Nishikawa et al., 2019). Our results suggest that oral bacteria, including periodontopathic bacteria, can affect gastric and gut microbiota and influence postoperative health after gastrectomy. In contrast, appropriate oral interventions may alter the bacterial environment in the gastrointestinal tract (Nishikawa et al., 2021). Because the oral cavity is more directly accessible than the stomach and gut, it may theoretically be possible to control the gut microbiota by controlling the oral microbiota. Therefore, the interactions among the gastrointestinal microbiota as a whole should be further elucidated.

This study has certain limitations. First, a relatively small sample size owing to the collection of oral, gastric, and gut samples simultaneously at two time points (before and 6 months after gastrectomy for gastric cancer), despite this being a strength of the study. In addition, participants in this study were enrolled in a single institution. The lack of a validation cohort from another hospital in a different geographic area limits the generalizability of the findings. Multiple observations over a longer period will provide more insight, and the findings can be confirmed using a larger system. Second, the 16S metagenomic analysis used in this study emphasized species composition and community diversity while evaluating relative rather than absolute bacterial abundance. To obtain additional information regarding a specific bacterium, particularly regarding its functionality, more data, such as those from DNA sequence, long-read, shotgun, and omics analyses should be included. Furthermore, although this study focused only on bacteria of the gastrointestinal tract, a comprehensive analysis is needed to determine how not only bacteria but also viruses, fungi, archaea, and their metabolites change and relate to the entire gastrointestinal tract, including the oral cavity, stomach, and intestines, by gastrectomy. For this purpose, further functional studies, such as metabolomics or transcriptomics, are needed. Third, the method used in this study does not allow for an analysis that considers all confounding factors. In the selection of participants for this study, at the time of sample collection, patients receiving specific medications such as antimicrobials and those with specific comorbidities were excluded. However, after gastrectomy, patients may experience changes in factors that may affect the microbiota, such as diet, lifestyle, and weight loss, in addition to the effects of the surgery. In addition, in this study, the probiotic intake status of the participants before and after gastrectomy was not considered. Supplements containing certain Lactobacillus spp. and other species are known to affect the bacterial composition of the microbiota and may have influenced the results. The effects of these potential factors on changes in the microbiota should be elucidated as this may aid in the management of postoperative patients. Therefore, we believe this issue should be addressed in the future. In addition, this was an observational study, and the causal relationships remain unclear. The mechanisms underlying the results of this study require further investigation using animal experiments. Nonetheless, this is a preliminary study and provides a basis for characterizing alterations in the oral, gastric, and gut microbiota and their associations with gastrectomy.

## 5 Conclusion

We characterized the alterations in the gut microbiota due to gastrectomy and demonstrated the relationship between these changes and oral and gastric microbiota, thereby elucidating the interactions between the microbiota of the gastrointestinal tract and changes in the gastric environment. Further understanding of the interrelationships of the gastrointestinal microbiota in the context of alterations due to gastrectomy may provide a basis for addressing the clinical problems that occur in patients after gastrectomy from the perspective of the microbiota.

## Data Availability

The data presented in the study are deposited in the DNA DataBank of Japan (DDBJ) BioSample database under accession number: SAMD01578217-SAMD01578306.
