# Supplementary figures and images for: Gastrectomy-induced alterations in gut microbiota linked to changes in oral and gastric microbiota
Source: Front Microbiol. 2025 Jun 18;16:1599503. doi: 10.3389/fmicb.2025.1599503 (PMC12213597; doi:10.3389/fmicb.2025.1599503)

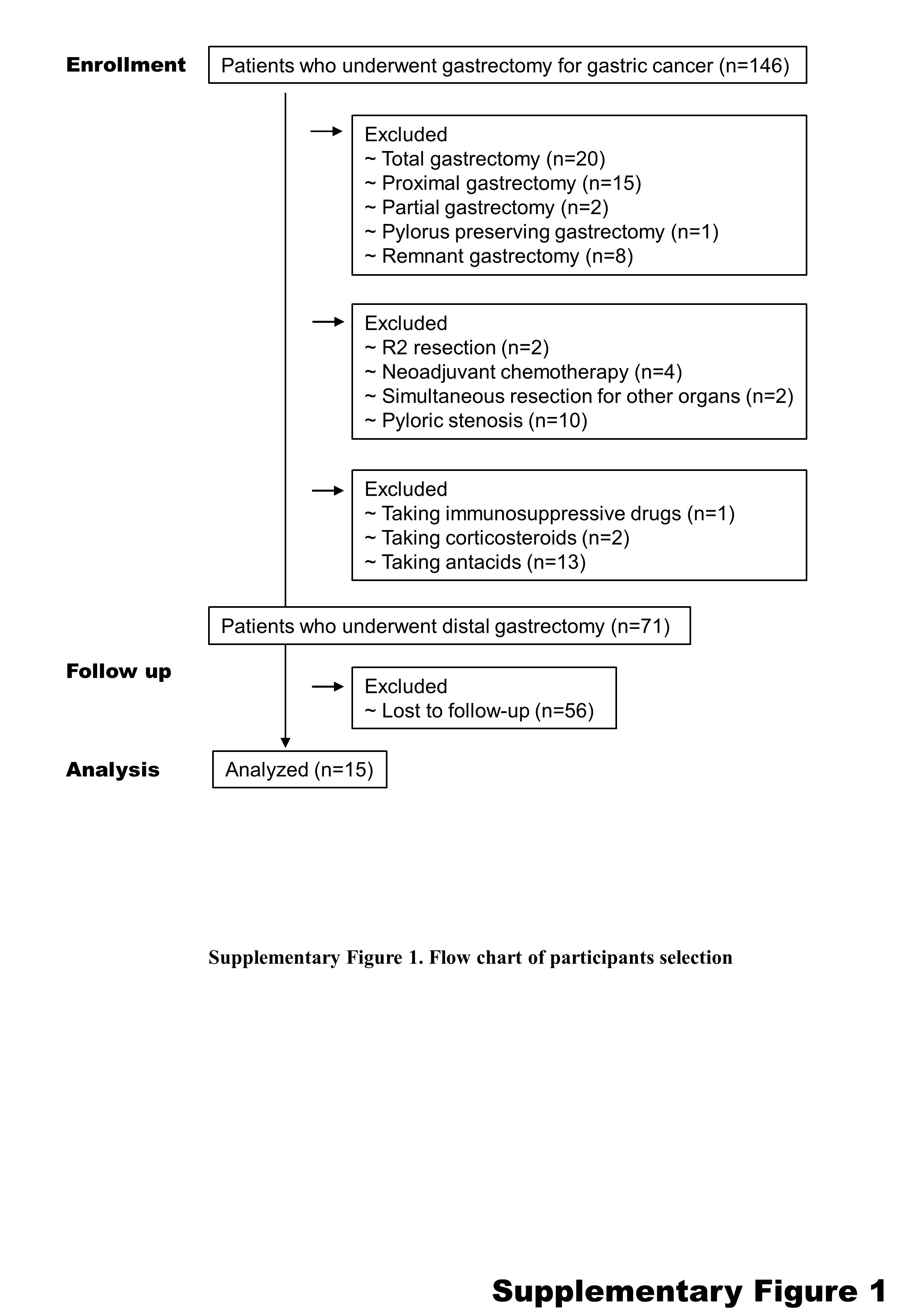

Supplement: Supplementary file 1 [file Image_1.jpeg]
